# Familial breast cancer: management of ‘lower risk’ referrals

**DOI:** 10.1038/sj.bjc.6603389

**Published:** 2006-10-03

**Authors:** D Young, L McLeish, F Sullivan, M Pitkethly, M Reis, D Goudie, H Vysny, G Ozakinci, M Steel

**Affiliations:** 1 Tayside Breast Cancer Family Clinic, Ninewells Hospital and Medical School, Dundee DD1 9SY, UK; 2 Community Health Sciences Division, University of Dundee Medical School, McKenzie Building, Kirsty Semple Way, Dundee DD2 4BF, UK; 3 Bute Medical School, University of St Andrews, St Andrews, Fife KY16 9TS, UK

**Keywords:** breast cancer, familial, clinical services, assessment, primary care

## Abstract

Up to 40% of referrals from primary care to ‘breast cancer family clinics’ prove to be of women whose assessed risk falls below the guidelines' threshold for management in secondary or tertiary care, despite recommendations that they should be screened out at primary care level. A randomised trial, involving 87 such women referred to the Tayside Familial Breast Cancer Service compared two ways of communicating risk information, letter or personal interview. Both were found to be acceptable to referred women and to their family doctors, although the former expressed a slight preference for interview. Only four women returned to their family doctors with continuing concerns about breast cancer. Nevertheless, understanding of information provided by either route was unsatisfactory, with apparent confusion about both absolute and relative risks of breast cancer. Substantial minorities appear to believe that they are at no increased risk at all, or even below the population level of risk, while others remain convinced that their personal risk has been underestimated. Family history record forms, completed by the referred women, preferably with the assistance of relatives, are crucial to full assessment of familial risk but one quarter of women referred to the Tayside Familial Breast Cancer Service currently do not complete and return these forms ahead of their clinic appointment. Further collaboration between primary care and the Breast Cancer Family Service is required to improve provision for concerned women whose risks fall below the threshold for special surveillance and to maximise effective use of the family history record form.

Guidelines published in the UK recommend that women concerned about a family history of breast cancer should be assessed first in a primary care setting and only those whose risk exceeds a specified threshold should be referred to specialist services for counselling, screening and possible intervention (Harper, 1996; SIGN, 1998; Eccles *et al*, 2000; Haites *et al*, 2000; NICE, 2004). In reality, however, general practitioners (GPs) find this ‘gatekeeper’ role difficult, both in the UK (Fry *et al*, 1999; Bankhead *et al*, 2001; Rose *et al*, 2001; Walter *et al*, 2001; Elwyn *et al*, 2002; Campbell *et al*, 2003) and elsewhere (Escher and Sappino, 2000). The proportion of referrals to breast cancer family clinics that fall below the required risk threshold has been reported as almost 25% in one large UK-wide survey (Wonderling *et al*, 2001). For Scottish clinics that figure is 30–40% (Wonderling *et al*, 2001; Holloway *et al*, 2004; Reis *et al*, 2006), the difference probably being explained by greater ease of extension and verification of reported family histories in Scotland through access to the National Cancer Registry and to public records of Births, Marriages and Deaths (Collyer and DeMay, 1997; Brewster *et al*, 2004). The term ‘low risk’ is commonly used as shorthand, even in some official guidelines, to define those falling below the threshold, although such women are generally at greater risk (up to 1.7 times higher) than women of comparable age with no family history of breast cancer.

From its inception in 1994, the Tayside familial breast cancer clinic (TFBCC) has been a multidisciplinary service run and staffed jointly by the Departments of Genetics, Breast Surgery and Radiology. Before this study began, all women referred to the TBFCC were offered an appointment, even if the family history appeared to place them at ‘low’ risk. When that assessment was confirmed, no further follow-up would be arranged, although clinical examination (sometimes supplemented by mammography), was offered before discharge. Inappropriate inclusion of these ‘lower risk’ women in surveillance programmes probably does not represent cost-effective use of limited resources (Reis *et al*, 2006). However, the need to *‘convey to individuals, especially those at low risk, accurate information in a sensitive and supportive manner’* must still be met (Harper, 1996). We report the outcome of a randomised trial of two approaches to this objective, together with difficulties encountered and possible solutions.

## METHODS

Before the start of the study, which ran for 30 months from August 2000, all General Practices in the Tayside catchment area were issued with a breast cancer genetics ‘information pack’ developed by the Cancer Research Campaign (Watson *et al*, 2001) and modified, with the agreement of the authors, to refer specifically to Scotland. They were also informed by letter and through presentations at GP study days, and other locally arranged seminars, about the functions of the breast cancer family clinic and the planned trial. Continuing information was provided through the website of the Department of Surgical Oncology, University of Dundee, and through reports in the Newsletter of the Tayside Primary Care research Network.

With approval from the Tayside Research Ethics Committee, all women referred to the TBFCC were invited to participate in a trial, comparing provision of information about their familial risk by letter or by personal interview. This would apply only if they were judged to fall below the 1998 SIGN guidelines threshold for inclusion in a regular surveillance programme (SIGN and NICE thresholds are very similar). All referred women also received a standardised Family History Record form with a request to complete it as far as possible, preferably in consultation with relatives, and to return it as the first stage in their risk assessment. That form, together with the GP referral letter, augmented as appropriate in each case (and with relevant informed consent) by checking hospital records, Cancer Registry entries and Registrar General's Records of Births, Marriages and Deaths, provided the basis for a consensus decision, taken by the specialist genetics staff, whether to offer an appointment to the multi-disciplinary counselling/surveillance clinic. Enrolment in the trial thus required written informed consent, a completed Family History Record form and a clear decision that familial risk was below threshold level.

Women who met these criteria were randomised by a genetics associate (using computer-generated random numbers) to receive the information in a personalised letter or to attend the genetics department for an interview (with a genetics associate or nurse specialist) which gave an opportunity for questions to be asked and answered but did not include clinical breast examination or mammography. This was followed-up by a personal letter summarising the discussion. All letters included the information that, despite being below ‘threshold’ level, risk of breast cancer was still real. Women should therefore remain ‘breast aware’, report any breast symptoms promptly to their GP, notify TFBCC of any change in their family history of breast/ovarian cancer and participate in the National Breast Screening Programme from age 50. Letters were copied to the referring GP. The two subgroups were well matched for age and social class, the latter being assessed by postcode.

Three months after the letter or interview, participants in the trial were asked to complete and return a ‘Satisfaction Questionnaire’, based on the instrument used in the Wales ‘TRACE’ study (Brain *et al*, 2000; Gray *et al*, 2000). The constituent elements are listed in Table 1. They included standardised and validated measures of psychological health as well as specific reactions to the service received.

Eighteen months after the end of the trial period, all GPs who had referred patients included in the trial were asked to complete and return a short questionnaire to evaluate the service provided and specifically to gather information on whether the women had returned to their family doctors with continuing or fresh concerns about breast cancer.

For data analysis Statistical Package for Social Sciences (SPSS™) software was used.

## RESULTS

**Figure 1:**
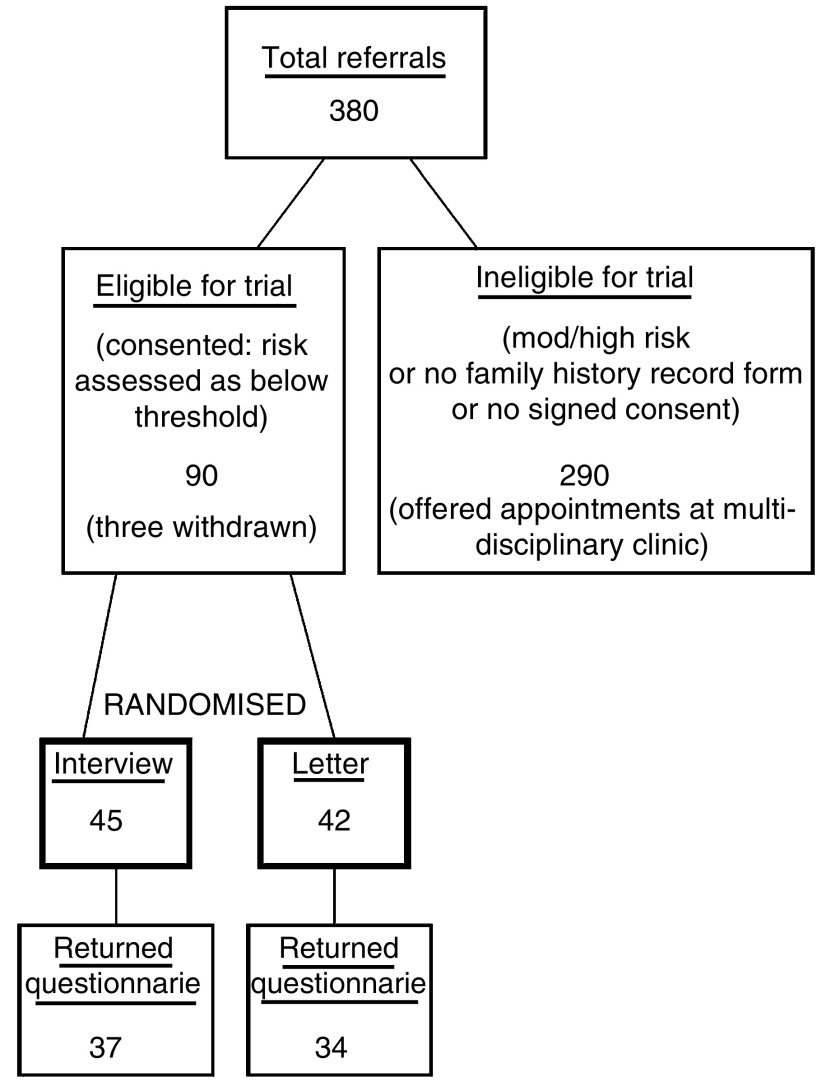
Distribution of referrals to the Tayside Familial Breast Cancer Service and recruitment to randomised trial.

During the study period, 380 women were referred to the TBFCC. Three quarters of these referrals came directly from Primary Care, the remainder being referred from the symptomatic breast clinic. Two hundred and eighty-one (74%) returned their ‘Family History Record’ form but 99 (26%) failed to do so, even after a personal reminder letter. Around half of these brought the form with them when they attended the clinic. Of those who did return the form, 64 (23%) did not give written consent to enter the trial. Only 18 of the 64 actively declined. The remainder simply did not return the consent document or returned it unsigned. Again, many brought the signed form to the multidisciplinary clinic but, even if ‘low’ risk status was confirmed, they could not then be randomised. There were therefore 217 women eligible for the study and, after full assessment as described above, 90 of these (41.5%) were judged to be below the guideline threshold level of genetic risk. They were therefore randomised to ‘letter’ (43) or ‘interview’ (47). Three were subsequently withdrawn; one, assigned to the ‘letter’ group, was found to have a cancer on initial examination at the symptomatic breast clinic (she had been referred there because of vague breast symptoms but family history had been mentioned in the GP letter and onward referral to the cancer family clinic had already been arranged, although no ‘low risk’ letter was actually sent). The other two provided, at interview, new information shifting them to the ‘moderate’ risk category. Among the other 45 ‘low risk’ women interviewed, five gave new information requiring additional checks on family history and three mentioned breast symptoms that led to investigation by a breast surgeon but all remained in the ‘low’ risk category and no significant breast pathology was found. These data are summarised in Figure 1.

Seventy-one of the 87 randomised study patients (81.6%) completed and returned the 3 month ‘satisfaction’ questionnaire. The 87 patients had been referred by 82 GPs, of whom 64 (78%) responded to the follow-up questionnaire (with replies relating to 69 patients – 79%). Analyses of the responses are presented below.

### Patient-completed satisfaction questionnaire

Independent samples *t*-tests were applied to all comparisons.

‘Concerns about breast cancer’ (6 items). Interitem correlations were good so the six were averaged to generate an index of breast cancer concerns. No difference was found between ‘letter’ and ‘face-to-face’ groups, *t*(69)=−0.636, *P*=0.527.

‘Actions since referral’ (10 items). Correlations among items varied but all were significant. No differences between ‘letter’ and ‘face-to-face’ groups were significant.

‘Experiences since referral’ (12 items). Correlations between items were all significant so scores were averaged. The difference between averaged scores for ‘letter’ *vs* ‘face-to-face’ groups did not reach significance, *t*(69)=−1.676, *P*=0.098.

‘Personal breast cancer risk estimate’. There was a significant difference between ‘letter’ group (mean=2.0) and ‘face-to-face’ group (mean=2.38), meaning that those receiving their information at interview perceived their risk to be slightly higher than those informed by letter, *t*(69)=−2.246, *P*=0.028.

‘Concern about personal breast cancer risk’. No significant difference was found between ‘letter’ and ‘face-to-face’ groups, *t*(69)=−0.705, *P*=0.483.

‘Population lifetime risk of breast cancer’. Respondents were invited to estimate population risk in two formats (see Table 1). For one of these, five responses were missing. There was no difference between ‘letter’ and ‘face-to face’ groups for either item, *t*(64)=.424, *P*=0.673 and *t*(69)=0.194, *P*=0.846.

‘Your own lifetime risk of breast cancer’. Again, this question was posed in two formats. Five responses were missing for one of these. No significant differences between the trial groups were found for either format: *t*(64)=1.036, *P*=0.304 and *t*(69)=−0.249, *P*=0.804. Both population and personal risk estimates were, however, often wildly inaccurate and there were poor correlations between estimates expressed in the two different formats by the same respondent.

‘Satisfaction with the process’. For five of the 12 items in this set of questions, the ‘face-to-face’ group expressed significantly higher levels of satisfaction than the ‘letter’ group (*P* range 0.020–0.001) although the mean scores for the ‘letter’ group were in the ‘quite satisfied’ to ‘very satisfied’ range.

‘General Health Questionnaire’. Scores for each of the four subgroups were summed and a *t*-test carried out on each. None of the differences between ‘letter’ and ‘face-to-face’ groups were significant.

In addition to the above quantifiable responses, women were invited to provide free text answers to open ended questions about their reactions to the information given. The majority either left these text boxes blank or indicated that they were content with the process. However, 7 (4 from the ‘letter’ and three from the ‘interview’ group) made statements indicating that they now believed they were at very low risk of breast cancer – possibly less than that of the general population. (*‘I was happy to learn that it doesn't run in families and I am more relaxed about everything’. ‘Quite happy that I am at considerably low risk’. ‘Happy to know my risks are not increased by my mother having developed breast cancer’.*) A further seven (four ‘letter’, three ‘interview’) took the opposite view and clearly did not accept the judgement that they were at the lower end of the genetic risk spectrum (‘*I cannot feel reassured by the response I received’. ‘I don't know if I believe what you told me; you are giving me a result from statistics which can prove whatever you want to prove. You are not giving me medical facts’.*)

### GP questionnaires

All but two of the 64 GPs declared themselves completely satisfied with the management of their individual patients. One had some reservations because of the time that elapsed (several months) between his referral and communication of the low assessed risk. Another was dissatisfied because he had no record of the outcome of his referral (although a copy letter had been sent to him).

When asked how they felt about a policy of evaluating risk before offering any clinic appointment and of declining appointments by explanatory letter for those judged to be below ‘threshold’ risk level, 46 of 64 respondents (72%) had no reservations. Seventeen had some reservations and one had serious reservations; where specific reasons were given, these related to the anticipated difficulty for some patients in completing a standard Family History Record form.

No patient had complained to the GP about the way in which their risk status had been assessed or communicated and only four had returned to the GP with concerns about breast cancer in the 18–48 months since receiving their clinic report. Two had fresh complaints of breast discomfort, which were investigated in the regional breast unit and two had simply wished to discuss the information from the genetics clinic. No breast cancers were recorded at that point but one other patient has subsequently developed invasive breast cancer at age 62 years.

## DISCUSSION

Our findings show that in deriving the best possible estimate of future cancer risk, face-to-face interview adds little to a detailed family history form (particularly if completed as a collaborative project by several relatives) verified and extended by access to hospital, Cancer Registry and Registrar General's records. Communication of risk information and its implications, however, still presents difficulties.

Patients, whether informed by letter or by interview, seemed to be very uncertain of their actual risk level some 3 months later, at least when invited to give it a numerical value. The discrepancies between two alternative ways of presenting that information may suggest a lack of clarity in the questions or difficulties with numerical notation. Communication of risk in the setting of a breast cancer family clinic is well recognised as a problem area, with no method of communication proven to achieve accurate understanding (Watson *et al*, 1998; Cull *et al*, 1999; Braithwaite *et al*, 2004; Lobb *et al*, 2005). Furthermore, the free text comments from a number of respondents showed that, despite scrupulous avoidance of the term ‘low risk’ in oral and written communications from the clinic, some feel inappropriately reassured, to the extent of believing their risk may be below that of the general population. Conversely, others evidently cannot accept that their risk does not justify special screening (ineligibility for mammography being resented). Overall, the mean level of satisfaction with the process was acceptable, lying between ‘quite satisfied’ and ‘very satisfied’, although the scores for the ‘interview’ group were significantly better than for those receiving the information by letter. There were no significant differences between ‘letter’ and ‘interview’ in subsequent measures of cancer worry, nor of general psychological health. Despite the recorded preference for face-to-face communication, only four women had returned to their GP with concerns about breast cancer and two of these had been in the ‘interview’ group.

The GP questionnaires revealed no preference for either method of delivering the risk evaluation and, in general, a process whereby referred patients were assessed without necessarily being seen in person at a genetics clinic was considered acceptable.

## CONCLUSION

Given that there must be a threshold level of risk below which clinical and mammographic screening cannot be offered, some disappointment, and hence dissatisfaction with the service is inevitable. Studies, including one from Scotland (Julian-Reynier *et al*, 1996; Lalloo *et al*, 1998; McLeish, 2003), have shown that women with a family history of breast cancer place access to regular mammography as their highest priority and indeed, so long as that is provided, they are content to forego specialist genetic assessment and counselling (Brain *et al*, 2000). The counterpart of that is that some women, when told their risk falls below guidelines' threshold, will resent exclusion from a surveillance programme. Several women used the free text boxes to express disappointment that they had not received any screening or ‘professional examination’ or to comment that they were relieved to know they would receive regular mammography from age 40 years (through a workplace or private healthcare scheme). Our findings in this regard are consistent with those of Scott *et al.* (2005) who interviewed a selected group of eight women judged to be at below threshold risk level and noted that several of them wished to have their risk ‘up-rated’ so that they would become eligible for screening.

No procedure, short of providing universal access to regular mammography, is likely to satisfy all women and any method of risk assessment will prove flawed in individual cases, as we have found. Nevertheless, our findings suggest that for most women at the lower end of the familial risk spectrum, communication of this information does not require a personal interview. A letter can be an adequate substitute. There is still scope for improvement without incurring unjustifiable costs. For example, the letter might include an invitation to contact the Genetics Centre to discuss continuing concerns. There may be a place for group sessions with specialists such as dieticians, counsellors and breast care nurses, where information on risk reducing ‘lifestyle’ modification may be offered and questions can be answered. Particular attention must also be given to methods of explaining risk, perhaps making use of high quality, specifically designed leaflets. Generic literature available from patient support groups, cancer charities and other clinics may also be useful but it will be important to harmonise the information they contain (Ozakinci *et al*, 2006).

The concern raised by several GPs about women who find it difficult to complete a Family History Record needs to be addressed. The fact that noncompletion of this form previously guaranteed access to the multidisciplinary clinic was perhaps a disincentive to its proper use and insistence on return of the form as a precondition for access to the cancer family service will almost certainly improve compliance. Rather than simply rejecting referrals in the absence of a completed form, however, it seems preferable to enlist the support of the primary care team in establishing why it has not been returned and in assisting those with genuine difficulties to collect and collate whatever family information may be available.

## Figures and Tables

**Table 1:** Components of patient ‘satisfaction’ questionnaire

Element 1	Concerns about breast cancer Six questions, based on the breast cancer worry scale (Lerman *et al* (1991)). Each has four possible responses (tick boxes) indicating degree of worry
	
Element 2	Actions since receiving risk assessment Twelve questions about possible adverse effects on behaviour. Each has four possible responses indicating degree of adverse effect
	
Element 3	Experiences since receiving risk assessment Ten questions about possible positive effects on behaviour. Each has four possible responses, indicating degree of positive effect
	
Element 4	Understanding of breast cancer risks Eleven questions, five about perception of own risk, two about perception of general population risk, 1 about motivation for seeking risk assessment, three about remaining concerns and sharing them with family members. Answers were mainly options to tick or circle but own and population risk perceptions were presented both as a list of possible odds (‘Inevitable ‘, ‘ 1 chance in 2’, ‘1 chance in 3’ through to ‘1 chance in 100’) and also on a linear percentage scale, from 0 (‘definitely will NOT get it’) through to 100% (‘definitely WILL get it’)
Element 5	Experiences of the interview or written communication(s) with the clinic Twelve questions about amount of information given, whether it was understandable, whether questions were answered, whether risk given differed from expected, whether the process had helped in coping with perceived risk and whether the timescale for the process had been acceptable. Responses were mainly in the form of tick boxes with four options but free text space was included for expression of opinions
Element 6	General Health Questionnaire Twenty-eight item format with four subsections (Goldberg and Williams (1988))
